# Fetal death after the introduction of COVID-19 mitigation measures in Sweden, Denmark and Norway: a registry-based study

**DOI:** 10.1038/s41598-022-25036-1

**Published:** 2022-11-30

**Authors:** Maria C. Magnus, Laura L. Oakley, Anne V. Hansen, Anne K. Örtqvist, Tanja G. Petersen, Laust H. Mortensen, Mette Bliddal, Anne-Marie Nybo Andersen, Olof Stephansson, Siri E. Håberg

**Affiliations:** 1 Centre for Fertility and Health, Norwegian Institute of Public Health, P.O. Box 222 Skøyen, 0213 Oslo, Norway; 2 Department of Non-Communicable Disease Epidemiology, London School of Hygiene and Tropical Medicine, London, UK; 3 Department of Public Health, University of Copenhagen, Copenhagen, Denmark; 4 Statistics Denmark, Copenhagen, Denmark; 5 Clinical Epidemiology Division, Department of Medicine, Solna, Karolinska Institutet, Stockholm, Sweden; 6 Department of Obstetrics and Gynaecology, Visby County Hospital, Visby, Sweden; 7 Open, Odense University Hospital, Odense, Denmark; 8 OPEN, University of Southern Denmark, Odense, Denmark; 9 Clinical Pharmacology, Institute of Public Health, University of Southern Denmark, Odense, Denmark; 10 Department of Women’s Health, Karolinska University Hospital, Solna, Stockholm, Sweden

**Keywords:** Medical research, Epidemiology

## Abstract

It remains unclear whether the rate of fetal death has changed during the COVID-19 pandemic. We assessed the impact of COVID-19 mitigation measures on fetal death in Sweden (449,347 births), Denmark (290,857 pregnancies) and Norway (261,057 pregnancies) using robust population-based registry data. We used Cox regression to assess the impact of the implementation of pandemic mitigation measures on March 12th, 2020, on miscarriage (fetal loss before gestational week 22) and stillbirth (fetal loss after gestational week 22). A total of 11% of 551,914 pregnancies in Denmark and Norway ended in miscarriage, while the proportion of stillbirths among 937,174 births across the three countries was 0.3%. There was no difference in the risk of fetal death during the year following pandemic mitigation measures. For miscarriage, the combined hazard ratio (HR) for Norway and Denmark was 1.01 (95% CI 0.98, 1.03), and for stillbirth, the combined HR for all three countries was 0.99 (95% CI 0.89, 1.09). We observed a slightly decreased risk of miscarriage during the first 4 months, with an HR of 0.94 (95% CI 0.90, 0.99) after lockdown. In conclusion, the risk of fetal death did not change after the implementation of COVID-19 pandemic mitigation measures in the three Scandinavian countries.

The COVID-19 pandemic has had broad social, economic and health-related impacts^1,2^. It was suggested early on that pandemic mitigation measures could influence the rate of pregnancy outcomes; however, whether this is the case is controversial due to inconsistent findings across countries^3^. Some studies report evidence of an increase in stillbirths during the first wave of the pandemic^4–9^, while other studies found no evidence of a difference^10–17^. A meta-analysis concluded that there was significant evidence of an increase in stillbirths after pandemic lockdown^18^. The majority of the existing studies had a limited sample size, with fewer than 30 stillbirth cases after the onset of the pandemic^4,6,10–12^. The largest study to date used information on more than 400,000 births during the pandemic identified through National Health Service hospital admissions in England and indicated no difference in the rate of stillbirth after the start of the pandemic^19^. There are no existing studies that compare the rate of miscarriage following pandemic lockdown to rates during the prepandemic period.

The Scandinavian countries, Sweden, Denmark and Norway, are similar with regard to their universal healthcare, levels of income inequality, and fertility patterns. When the World Health Organization declared that COVID-19 had reached a pandemic level (March 13, 2020), the number of infected persons was relatively low in all three Scandinavian countries. Denmark and Norway initiated strict pandemic mitigation measures in mid-March in an attempt to avoid the high burdens on the health-care systems observed across several other European countries, while Sweden followed a less strict approach^20–22^. There were clear changes in the behaviour of the populations in all three countries from mid-March, resulting in a decreased use of public transportation, less workplace commuting and more time spent at home^23^. Behavioural measures indicated that the strict lockdowns of Denmark and Norway resulted in more drastic behavioural changes than in Sweden^24^. We hypothesized that the pandemic mitigation measures could plausibly decrease the risk of fetal death, as pregnant women were likely to have less exposure to infections^25,26^.

The objective of this study was to assess the impact of COVID-19 mitigation measures on the risk of fetal death using national registry-based data from Sweden, Denmark and Norway.

## Materials and methods

### Study population

We extracted information on pregnancies registered with a live birth, stillbirth or miscarriage between January 2017 and March 2021. In Denmark and Norway, information was available from national Medical Birth Registers and National Patient Registers and included all pregnancies resulting in any contact with specialist health-care services (also referred to as secondary/tertiary). The data from Denmark and Norway therefore included all births (live and stillbirths), in addition to all recognized miscarriages resulting in contact with specialist health-care services. This is likely to include the overwhelming majority of recognized first-trimester miscarriages and all second trimester miscarriages. In Norway, the exact gestational age of the first-trimester miscarriages occurring before 12 completed gestational weeks was not available. Based on registrations in Denmark, the median gestational week of the registered first-trimester miscarriages was 8 weeks (interquartile range 6, 9). From the Swedish Pregnancy Register, we retrieved information on all births at ≥ 22 gestational weeks during the same time period; in Sweden, 92% of births are included in the national register (18 of 21 Swedish regions). We therefore had information on all registered pregnancies for Denmark and Norway, while we only had information on births for Sweden. Further details of the data sources are listed in the appendix (supplementary online methods). This study was approved by the Regional Committee for Medical and Health Research Ethics of South/East Norway (#141135) and the Swedish Ethical Review Authority (approval numbers: dnr 2020-01499, dnr 2020-02468, dnr 2021-00274). Denmark, the study was registered with the Danish Data Protection Agency via the University of Southern Denmark (reg. no. 364 20/17416) and via Statistics Denmark. Each committee provided a waiver of consent for participants. The ethical committees mentioned above in the three Nordic countries are grounded in foundational ethical principles embodied in the Declaration of Helsinki of 1964 and its subsequent revisions and the Belmont Report.

### Pandemic mitigation

Although the intensity and timing of COVID-19 mitigation measures differed between the three countries, the majority of measures were introduced around March 12th, 2020 (Table 1). Thus, March 12th, 2020, was taken as the common date for the introduction of pandemic mitigation measures (“lockdown”) across all three countries. The exposure of interest was therefore pregnancy days after this date. Notably, Norway and Denmark had an overall very similar approach to the implementation of pandemic mitigation measures. These two countries implemented similar strict measures around the same time. In contrast, Sweden had fewer and less strict pandemic mitigation measures. The details of the specific measures are described in Table 1.

**Table 1:** Summary of early COVID-19 mitigation measures in Norway, Sweden and Denmark.

	Sweden	Denmark	Norway
Daycare and primary schools closed	n/a	March 16	March 12
High school and universities closed	March 17 (recommendation)	March 13	March 12
Restrictions on gathering	March 11 (500+) March 27 (50+)	March 11 (100+) March 17 (10+)	March 12
Home office recommendations	March 16 (recommendation to use home office)	March 13 (Non-essential workers in public sector ordered to stay home, private sector urged to permit use of home office)	March 10 (recommendation to use home office)
Non-essential business closed		Some closures from March 18, including restaurants/bars	Some closures from March 12
Recommendations to stay at home	March 16 for over 70 s March 19 Avoid unnecessary travels	March 11 restrict public transport and unnecessary travels	March 12 Avoid public transport and unnecessary travels, March 19 not allowed to spend night in vacation homes outside home county
Regulation of internal (domestic) movement	March 19	April 9	March 12
International travel restrictions	March 14 Advice against all unnecessary international travels, isolation and get tested if symptoms after arrival to Sweden	March 11 (flights from high-risk areas cancelled) March 14 (all borders closed)	March 13 Recommendations to avoid all unnecessary international travel, mandatory quarantine when arriving Norway, isolation if symptoms
Public events cancelled	March 12	March 13	March 12

### Fetal death

We defined fetal death as miscarriage or stillbirth based on gestational age, where fetal deaths with a gestational age ≥ 22 weeks were defined as stillbirths. In Denmark, pregnancies were identified using the Medical Birth Registry (including fetal deaths after 22 completed gestational weeks and all live births) and the national Patient Registry (for fetal death before 22 weeks). For Norway, the Medical Birth Registry included all pregnancies ending after 12 completed gestational weeks, while information on pregnancies ending prior to this time was obtained through the National Patient Registry. In Sweden, we obtained information on deliveries after 22 completed gestational weeks from the Pregnancy Register. Further details on the identification of pregnancies across the three different countries are available in the supplemental methods. We examined the risk of miscarriage only in Denmark and Norway, while the risk of stillbirth was examined in all three countries.

In Denmark, information on all pregnancies was available from the National Patient Registry and the Danish Medical Birth Register^27,28^. The Danish Medical Birth Register includes all births up to December 31, 2018, but only a proportion of births from the first quarter of 2019. Therefore, from January 1, 2019, we identified live births based on registrations of International Classification of Disease version 10 (ICD-10) codes Z38 and O80-84, and stillbirths based on registrations of ICD-10 code P95 in the National Patient Registry. Any registration of stillbirths with a birthweight of < 500 g and where gestational age was missing or less than 22 weeks was reclassified as miscarriages. Miscarriages were further identified using ICD-10 codes O02- “spontaneous abortion” and O03- “other abnormal products of conception”. In Norway, information on all pregnancies ending after 12 completed gestational weeks was available from the Medical Birth Registry of Norway^29^. All pregnancies recorded in the birth registry are designated as either having resulted in a live birth or a fetal death. We distinguished fetal deaths according to whether they were a miscarriage or a stillbirth based on information on birthweight and gestational age. We defined a fetal death as a miscarriage if the gestational age was < 22 completed weeks and the birthweight was < 500 g, while a fetal death with a gestational age ≥ 22 completed gestational weeks or a birthweight ≥ 500 g was classified as a stillbirth. Information on miscarriages prior to 12 completed gestational weeks was available from the patient registry^30^. As in Denmark, we used the ICD-10 codes O02 and O03 to identify miscarriages. In Sweden, we had information on all deliveries (live and stillbirths) after 22 completed gestational weeks from the Swedish Pregnancy Register. This quality register was initiated in 2013 and includes 92% of all births in Sweden (18 of 21 regions).

### Statistical analysis

Individual-level data were analysed for each country separately. We examined differences in the risk of miscarriage and stillbirth using Cox proportional hazards regression with the gestational day as the time metric. We chose to use a Cox regression to distinguish between the time of the pregnancy that was pre-pandemic as opposed to during the pandemic. To avoid oversampling of short pregnancies and fetal losses towards the end of the study period, we excluded all pregnancies that did not have the opportunity to reach 42 completed gestational weeks by the end of follow-up based on the best estimate of the start of pregnancy, prioritising ultrasound measurements where this was available. Exposure to pandemic mitigation measures (e.g., pregnancy days after March 12th 2020) was used as a time-varying exposure, derived using gestational day on the implementation of pandemic mitigation measures (March 12th, 2020). Women could therefore contribute both exposed and unexposed follow-up time if their pregnancy started before and ended after March 12th, 2020. We adjusted for calendar week as a discrete variable to account for seasonal variation in the risk of the outcomes of interest. We examined the validity of the proportional hazards assumption by visually inspecting the Schoenfeld residuals. The results from each country were subsequently combined using a random effects meta-analysis. As the individual-level datasets were housed at independent institutions in the three countries, it was not possible to conduct a pooled analysis of the individual-level data and test differences between the countries using an interaction term. Heterogeneity was instead assessed using the *I*^*2*^ statistic, calculated as 100% × (Q–df)/Q, where Q is Cochrane's heterogeneity statistic and df denotes degrees of freedom^31^. The main analysis examined whether the implementation of pandemic mitigation measures on March 12th, 2020, influenced the risk of fetal death during a 12-month follow-up period. Secondary analyses examined more immediate effects of the implementation of the pandemic mitigation measures during the 2, 4 and 6 months following March 12th, 2020. Analyses were performed using Stata version 16 (Statacorp, Texas).

## Results

A total of 449,347 births were recorded in Sweden during the study period, while 290,857 pregnancies (257,733 births) were recorded in Denmark, and 261,057 pregnancies (230,094 births) were recorded in Norway. The overall proportion of miscarriage among all pregnancies during the follow-up period was 11.4% in Denmark and 11.9% in Norway. Among all births, the proportion ending in stillbirth was 0.3% in all three countries. The proportion of stillbirth and miscarriage remained relatively stable in all three countries during the prepandemic study period.

### Trends in miscarriage in Denmark and Norway

**Figure 1:**
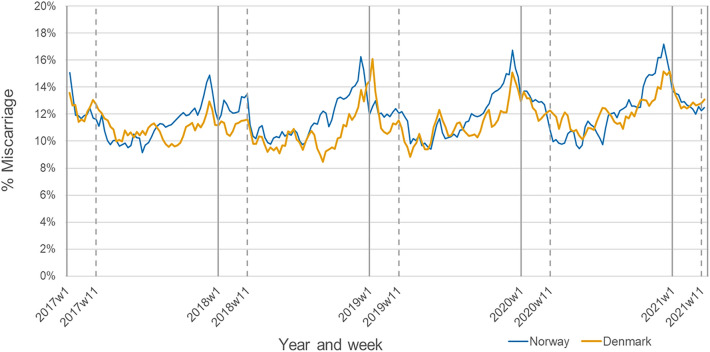
Proportion of pregnancies ending in a miscarriage in Denmark and Norway between January 2017 and March 2021. Calendar week 11 corresponds to the week of March 12th 2020. Sweden was not included in the analysis because the Swedish Pregnancy Register only includes deliveries after 22 completed gestational weeks.

**Figure 2:**
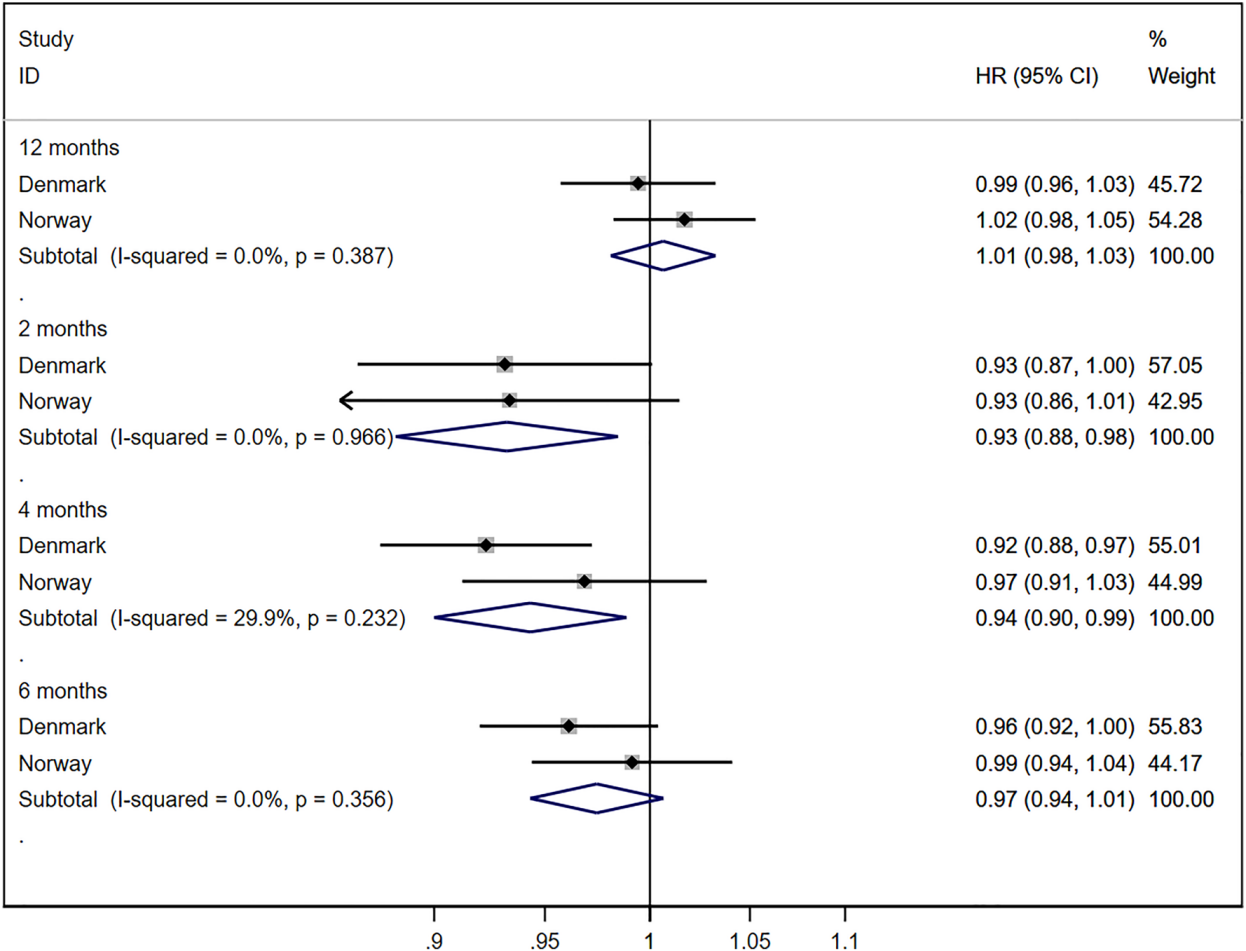
Estimates of the change in miscarriage in Denmark and Norway after March 12th, 2020. Sweden was not included in the analysis because the Swedish Pregnancy Register only includes deliveries after 22 completed gestational weeks. The hazard ratios are adjusted for calendar week at conception.

The proportion of pregnancies ending in a miscarriage was highest during the peak winter months and lowest during summer (Fig. 1). These seasonal variations were observed for all calendar years in both Denmark and Norway. The survival analysis indicated no increased risk of miscarriage during the 12 months following the implementation of the pandemic mitigation measure, with a combined HR of 1.01 (95% CI 0.98, 1.03) and no evidence of heterogeneity between countries (I^2^ 0%, *p* value 0.39) (Fig. 2). We observed a modestly decreased risk of miscarriage during the first 2 months (combined HR 0.93; 95% CI 0.88, 0.98) and first 4 months (combined HR 0.94; 95% CI 0.90, 0.99) following the implementation of the pandemic mitigation measures (Fig. 2). A corresponding decreased risk was not observed for the 6-month time window.

### Trends in stillbirth in Denmark, Norway and Sweden

**Figure 3:**
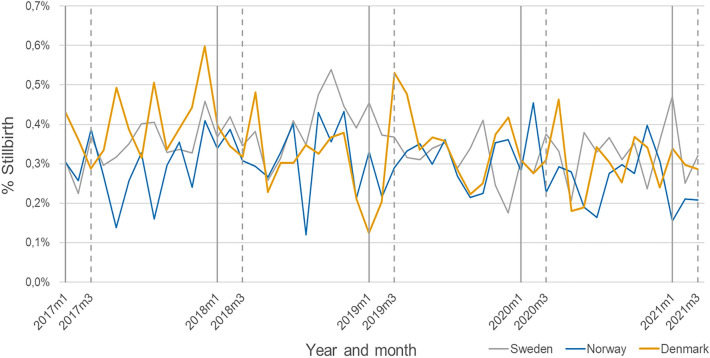
Proportion of deliveries ending in a stillbirth in Sweden, Denmark and Norway between January 2017 and March 2021. Calendar week 11 corresponds to the week of March 12th 2020.

**Figure 4:**
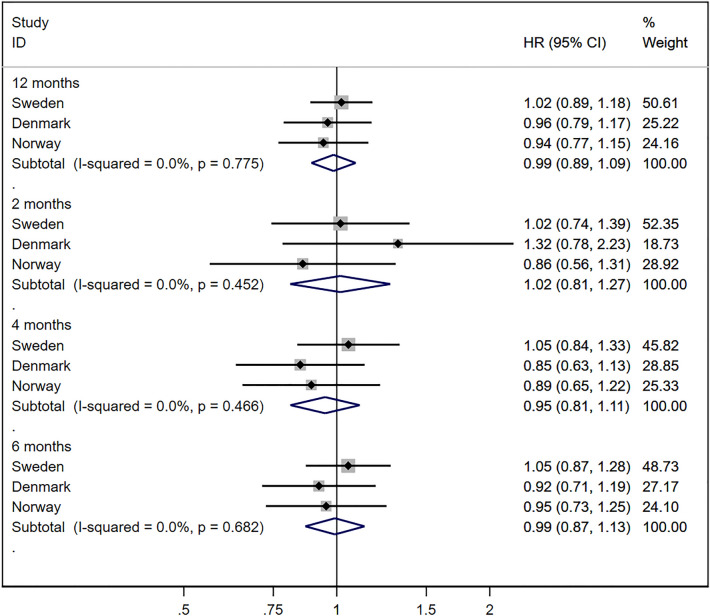
Estimates of the change in stillbirth in Sweden, Denmark and Norway after March 12th, 2020. The hazard ratios are adjusted for calendar week at conception.

Compared to seasonal variations in miscarriage, seasonal trends in stillbirth were much less pronounced (Fig. 3). There was no increased rate of stillbirth during the 12 months following the implementation of pandemic mitigation measures, with a combined HR of 0.99 (95% CI 0.89, 1.09), and no heterogeneity in the associations across countries (I^2^ 0%, *p* value 0.78) (Fig. 4). Similarly, there was no indication of more short-term impacts of the pandemic mitigation measures during the first 2 (combined HR 1.02; 95% CI 0.81, 1.27), 4 (combined HR 0.95; 0.81, 1.11) and 6 (combined HR 0.99; 95% CI 0.87, 1.13) months’ time windows.

## Discussion

Using national registries from Sweden, Denmark and Norway, we found no increase in fetal death after the implementation of COVID-19 pandemic mitigation measures across three Scandinavian countries. We observed a modest decreased risk of miscarriage during the first 4 months following March 12th, 2020.

Some existing studies suggest an increase in stillbirths following COVID-19 pandemic mitigation measures^4–9^, but the evidence is inconsistent^10–17^. As the majority of the existing studies have a very small sample size (with between 20 and 140 cases of stillbirth after lockdown), there is a high degree of uncertainty in the estimates. A meta-analysis of 6 studies suggested an increased risk of stillbirth during the first wave of the pandemic, with a combined incidence rate ratio of 1.33 (95% CI 1.04, 1.69)^18^. Differences in the findings across studies could be due to variations in the sample size, the country income level, financing of the health-care system, and the extensiveness of pandemic mitigation measures. Two of the previous studies included in the meta-analysis had ~ 140 cases of stillbirth after lockdown^5,7^, while the rest of the studies had only approximately 20 cases of stillbirth after lockdown or less^4,6,10–12^. The largest study to date (more than 130,000 births and 543 cases of stillbirth after lockdown) from the UK had a clear null finding, similar to that observed across the three countries in our study, with a risk of 0.36% during the pandemic versus 0.37% during the prepandemic period (*p* = 0.16)^19^.

To our knowledge, studies examining the change in the risk of miscarriage after the implementation of pandemic mitigation measures are lacking.

There are several well-known risk factors for miscarriage^32–35^ and stillbirth^36–38^, although understanding of the biological mechanisms remains inadequate. Our findings are reassuring given that general access to health care among pregnant women was reduced during the early stages of the pandemic. There are a number of potential explanations for how pandemic mitigation measures could plausibly contribute to a modest decreased risk of miscarriage. For example, certain infectious diseases are associated with an increased risk of miscarriage^26^, and a reduced number of social contacts (as a result of pandemic restrictions) may have led to fewer infections (not only COVID-19) among pregnant women. It is also possible that pandemic restriction measures resulted in less physical stress, which again could have reduced women’s risk of miscarriage, although the impact on work-related physical strain on the risk of miscarriage is likely to be modest^25^. However, the fact that we observed this modest decreased risk of miscarriage only during the first months after implementation of pandemic mitigation measures might also indicate that this modest reduction is unlikely to be causal. For example, if a larger proportion of miscarriages was seen only in primary care (and not referred to specialist health-care services) during this rather chaotic time, this could also explain this finding. The lack of a causal relationship between pandemic mitigation measures and risk of fetal death is further substantiated by the fact that we did not see any changes in stillbirth.

We studied more than one million pregnancies in the three Scandinavian countries from January 2017 through March 2021. The universal (free) health-care system in the Nordic countries and the mandatory registration of all contacts with the health-care system in the national registries ensured that we captured the majority of pregnancies during this period. For Norway and Denmark, all live and stillbirths (including home births) during the study period were included. For Sweden, we captured 92% of registered births (live and stillbirths), as information on approximately 8% of births is missing due to incomplete electronic data transfer in 3 of Sweden’s 21 counties^39^. The missing registrations did not depend on birth outcomes and would not bias associations. Information on early miscarriages registered in specialist health-care services in the Danish and Norwegian data at the population level is unique from an international perspective. For the Danish data, the introduction of new registration procedures during the study period may have led to inconsistencies in registration practices over time. Notably, we were only able to capture miscarriages resulting in contact with specialist health-care services. Very early miscarriages for which the woman did not seek health care or only contacted primary care services are therefore not captured. Based on previous estimates from the Norwegian general practitioner database, approximately ¼ of first-trimester miscarriages are only seen in primary care^40^. Therefore, our study will have underestimated the number of miscarriages. This could have contributed to our observation of a modest reduction in the number of miscarriages during the first months after the pandemic lockdown if a greater proportion of miscarriages were seen in the primary care during this time period because of a higher threshold for referring early miscarriages to specialist care during this early period.

The aim of our study was to assess the overall impact of pandemic mitigation measures on the rate of fetal death. We therefore did not include information on infection or vaccination for SARS-CoV-2. However, the current limited evidence indicates no strong evidence of an increased risk of fetal death among women who experienced SARS-CoV-2 infection during pregnancy^41–44^ or any evidence to support an increased risk of fetal death after vaccination^45–47^.

In conclusion, we observed no overall evidence of a change in the risk of miscarriage or stillbirth in the year following the implementation of pandemic mitigation measures across three Scandinavian countries.

## Supplementary Information

Supplementary Information.

## Data Availability

The individual-level data that support the findings of this study are not publicly available due to legal restrictions in all three Scandinavian countries. The analytical code can be accessed by contacting the corresponding author.
