# Supplementary material for: Fetal death after the introduction of COVID-19 mitigation measures in Sweden, Denmark and Norway: a registry-based study
Source: Sci Rep. 2022 Nov 30;12:20625. doi: 10.1038/s41598-022-25036-1 (PMC9709372; doi:10.1038/s41598-022-25036-1)
Supplement: Supplementary file 1 — Supplementary Information. [file 41598_2022_25036_MOESM1_ESM.docx]

**Online Methods**

*Identification of pregnancies*

Denmark

In Denmark, information on all pregnancies was available from the National Patient Registry and the Danish Medical Birth Register.^1, 2^ The Danish Medical Birth Register includes all births up to December 31 2018, but only a proportion of births from the first quarter of 2019. Therefore, from January 1 2019, we identified live births based on registrations of International Classification of Disease version 10 (ICD-10) codes Z38 and code O80-84 and stillbirths based on registrations of ICD-10 codes P95 in the National Patient Registry. Records of stillbirths with a birthweight of <500 grams and where gestational age was missing or less than 22 weeks were re-classified as miscarriages. Miscarriages were further identified using ICD-10 codes O02 and O03.

Norway

In Norway, information on all pregnancies ending after 12 completed gestational weeks was available from the Medical Birth Registry of Norway.^3^ All pregnancies recorded in the birth registry are designated as either having resulted in a live birth or a fetal death. We distinguished fetal deaths according to whether they were a miscarriage or a stillbirth based on information on birthweight and gestational age. We defined fetal deaths as a miscarriage if the gestational age was < 22 completed weeks and had a birthweight < 500 grams, while fetal deaths with a gestational age ≥ 22 completed gestational weeks or a birthweight ≥ 500 grams.

Information on miscarriages prior to 12 completed gestational weeks was available from the patient registry.^4^ We used ICD-10 codes O02-“spontaneous abortion” and O03-“other abnormal products of conception” to identify miscarriages. As a pregnancy might result in multiple records in the patient registry, we required that there be at least 42 days between these registrations to be counted as a new pregnancy. We further ensure that any registrations of these codes in the patient registry did not occur within the estimated duration of a pregnancy ending in the birth registry, and that they occurred at least 42 days after the end of a pregnancy registered in the birth registry. The miscarriages identified in the patient registry after this data cleaning procedure were therefore assumed to have occurred before 12 weeks as they would otherwise have ended in a registration in the birth registry.

Sweden

In Sweden, we had information on all deliveries (live and stillbirths) after 22 completed gestational weeks from the Swedish Pregnancy Register. This quality register was initiated in 2013, and includes 92% of all births in Sweden (18 of 21 regions).

References

1. Bliddal M, Broe A, Pottegård A, Olsen J, Langhoff-Roos J. The Danish Medical Birth Register. Eur J Epidemiol 2018;33:27-36.

2. Schmidt M, Schmidt SA, Sandegaard JL, Ehrenstein V, Pedersen L, Sørensen HT. The Danish National Patient Registry: a review of content, data quality, and research potential. Clin Epidemiol 2015;7:449-90.

3. Irgens LM. [Medical birth registry--an essential resource in perinatal medical research]. Tidsskr Nor Laegeforen 2002;122:2546-9.

4. Bakken IJ, Ariansen AMS, Knudsen GP, Johansen KI, Vollset SE. The Norwegian Patient Registry and the Norwegian Registry for Primary Health Care: Research potential of two nationwide health-care registries. Scand J Public Health 2020;48:49-55.
